# MXRA7 is involved in megakaryocyte differentiation and platelet production

**DOI:** 10.1097/BS9.0000000000000167

**Published:** 2023-07-05

**Authors:** Zhenjiang Sun, Benfang Wang, Ying Shen, Kunpeng Ma, Ting Wang, Yiqiang Wang, Dandan Lin

**Affiliations:** a Institute of Blood and Marrow Transplantation, National Clinical Research Center for Hematologic Diseases, Collaborative Innovation Center of Hematology, Jiangsu Institute of Hematology, The First Affiliated Hospital of Soochow University, Suzhou Medical College, Soochow University, Suzhou 215006, China; b Department of Clinical Laboratory, The Affiliated Jiangyin Hospital of Southeast University, Jiangyin 214400, China; c Wisdom Lake Academy of Pharmacy, Xi’an Jiaotong-Liverpool University, Suzhou 215123, China

**Keywords:** differentiation, megakaryocytes, MXRA7, platelet

## Abstract

Matrix remodeling is a critical process in hematopoiesis. The biology of MXRA7, as a matrix remodeling associated gene, has still not been reported in hematopoietic process. Public databases showed that MXRA7 expressed in hematopoietic stem cells, suggesting that it may be involved in hematopoiesis. We found that the amounts of megakaryocytes were lower in bone marrow and spleen from *Mxra7*^−/−^ mice compared with that from wild-type mice. Knock-out of MXRA7 also reduced the amount of platelet in peripheral blood and affected the function of platelets. Knock-out of MXRA7 inhibited hematopoietic stem/progenitor cells differentiate to megakaryocytes possibly through down-regulating the expression of *GATA-1* and *FOG-1*. Moreover, knockdown of MXRA7 in MEG-01 cells could inhibit the cell proliferation and cell apoptosis. Knockdown of MXRA7 inhibited the differentiation of MEG-01 cells and proplatelet formation through suppressing the ERK/MAPK signaling pathway and the expression of β-tubulin. In conclusion, the current study demonstrated the potential significance of MXRA7 in megakaryocyte differentiation and platelet production. The novel findings proposed a new target for the treatment of platelet-related diseases, and much more investigations are guaranteed to dissect the mechanisms of MXRA7 in megakaryocyte differentiation and platelet production.

## 1. INTRODUCTION

Matrix remodeling is a critical process occurred in hematopoietic microenvironment during differentiation and development of various blood cells,^1,2^ including platelets.^3^ It is recognized that the bone marrow (BM) extracellular matrix (ECM) is made up of various matrix proteins and soluble proteins, including collagens, fibronectin, fibrinogen, plasmin, cytokines, chemokines, enzymes, and so on. And many matrix proteins are involved in megakaryocyte (Mk) development. For example, previous studies have reported that type I collagen could stimulated hematopoietic stem cells (HSCs) to differentiate into megakaryocytic lineage.^4^ Fibronectin, fibrinogen, plasmin, thrombospondin-2 (TSP-2), and other BM ECM components have also been reported to affect Mk function and development, and proplatelet production.^3^ Conversely, Mk is involved in ECM deposition and reconstruction and can interact with extracellular components to maintain the physiology of hematopoiesis.^5^ However, the panorama of matrix remodeling in such conditions is not fully understood yet.

Matrix remodeling-associated (MXRA) genes family consists of eight numbers, MXRA1-MXRA8, which was co-expressed with genes involved in cell adhesion or matrix remodeling (matrix metalloproteinases, collagens, bone morphogenic proteins, etc).^6^ The members of this family were reported to be related with some critical physiological and pathological processes related to matrix remodeling. MXRA7 was firstly named in a bioinformatics study published in 2002 and belongs to the MXRA family. However, MXRA7 had been just mentioned in some studies without any purpose investigation into its biological functions except for our previous studies. MXRA7 was highly expressed in childhood acute lymphoblastic leukemia (ALL) and ovarian endometriomas.^7,8^ At first, our laboratory found a dynamic change of MXRA7 mRNA in inflammatory corneal disease models in adult mice.^9^ Then, we investigated the biological functions of MXRA7 using other models and demonstrated that MXRA7 was related to matrix remodeling that affected tissue damage or regenerative processes.^9–11^ Recently, we also found that the function of BM mesenchymal stem cells was related with MXRA7.^12^

Public databases showed that MXRA7 was expressed in HSCs, suggesting that it may be involved in hematopoiesis. Therefore, we deduced that MXRA7 played roles in the hematopoietic process. In this study, we used MXRA7 knock-out (*Mxra7*^−/−^) mice and wild-type (WT) mice to investigate the role of MXRA7.

## 2. MATERIALS AND METHODS

### 2.1. Experimental animals

The wild-type (WT) and *Mxra7*^−/−^ mice were generated by cross-breeding female and male *Mxra7*^+/−^ mice (C57BL/6N background) purchased from the Medical Research Council (MRC, Swindon, UK). The WT and *Mxra7*^−/−^ breeders were used for generating WT and *Mxra7*^−/−^ colonies respectively. The WT and *Mxra7*^−/−^ mice (aged 6–8 weeks) were used in this study. Specific pathogen-free C57BL/6N mice (aged 6–8 weeks) were purchased from Nanjing Biomedical Research Institute of Nanjing University (Nanjing, China). All mice were housed in specific pathogen-free facility in Soochow University. The animal experiments were approved by the Institutional Laboratory Animal Care and Use Committee of Soochow University (No.2016-075-1).

### 2.2. Cell line and human samples

MEG-01 cell line was obtained from ATCC (Manassas, Virginia) and cultured in 10% fetal bovine serum RPMI-1640 medium (Gibco, Grand Island, New York). Cells were cultured at 37°C and 5% CO_2_ in a humidified atmosphere. The bone marrow (BM) and peripheral blood mononuclear cell (PBMC) samples of healthy donors were obtained with written informed consent approved by the Ethical Committee of First Affiliated Hospital of Soochow University (No.2016-075-1).

### 2.3. Lentivirus transfection

Lentiviral transfection systems were purchased from Hanbio Biotechnology (Shanghai, China) to knock-down human MXRA7 in MEG-01 cells. The lentivirus of sh-MXRA7 and negative control (sh-NC) with GFP flag generated small interfering (si)RNA, and MXRA7 siRNA could target all three human MXRA7 transcripts. After infection lentivirus for 24 hours, MEG-01 cells were washed with 1× phosphate-buffered saline (PBS) and cultured for 48 hours in fresh complete medium. Then the GFP positive cells were sorted using flow cytometry and cell sorter (Melody, BD Bioscience, Franklin Lakes, New Jersey) to establish the MEG-01/sh-NC and MEG-01/sh-MXRA7 cell lines.

### 2.4. Flow cytometry analysis

Culture cells or single-cell suspensions prepared from femur, spleen or peripheral blood were analyzed using flow cytometry. The antibodies used for FACS staining including anti-mouse CD3 (100348), CD19 (152412), NK-1.1 (156504), CD11b (101226), TER119 (116212), Lineage (51-9003632), and CD41 (133916, 133904) antibodies were purchased from Biolegend (San Diego, California). Anti-human CD41 antibody (303706, 303710) was also purchased from Biolegend. Cells were harvested, stained with surface antibodies for 30 minutes and analyzed using NovoCyte flow cytometer (ACEA Biosciences, San Jose, California).

### 2.5. Hematoxylin and Eosin staining

The femur tissues and spleens from mice were fixed with 4% paraformaldehyde, dehydrated with different concentrations of ethanol, treated in xylene and then embedded in paraffin. Paraffin-embedded tissues were cut into 6-μm-thick sections. And the sections were deparaffinized, rehydrated, and stained with H&E for megakaryocytes (Mks) examination using a light microscope (Nikon, Tokyo, Japan).

### 2.6. Hematology analysis

The white blood cells (WBC), red blood cells (RBC), and platelets (PLT) in peripheral blood of mice were analyzed with Sysmex XP-100 Hematologic Analyzer (Sysmex Corporation, Japan).

### 2.7. Platelet preparation

The mice were anesthetized with pentobarbital sodium, and the blood was collected from inferior vena cava and anticoagulated with a 1:7 volume of acid-citrate-dextrose (ACD). The anticoagulated blood was centrifuged at 1100 rpm for 11 minutes to separate platelet-rich plasma (PRP). The platelets were collected from PRP by centrifugation at 3500 rpm for 2 minutes, then washed with CGS buffer, and resuspended in modified Tyrode’s buffer (MTB), and allowed to incubate at room temperature for 2 hours.

### 2.8. Mitochondrial membrane potential depolarization

Platelets were stimulated with 0.5 U/ml thrombin (THB, 386, Chrono-log, Havertown, PA) or 10 μM Adenosine diphosphate (ADP, 384, Chrono-log) for 30 min at room temperature. Mitochondrial membrane potential (ΔΨm) depolarization in platelets was detected by staining with 8 μg/mL JC-1 (C2006, Beyotime, Shanghai, China) and analyzed with a flow cytometer.

### 2.9. Platelet activation

Platelets were stimulated with 0.5 U/mL THB or 10 μM ADP for 30 min at room temperature, stained with anti-CD62P (12-0626-82, Thermo Fisher Scientific, Waltham, Massachusetts) and analyzed with flow cytometry.

### 2.10. Platelet adhesion

Platelet adhesion was performed using 96 well plates coated with type-I collagen (354236, Corning, Corning, New York) overnight at 4°C.^13^ Platelets were incubated in coated plates for 1 hour at 37°C. Washing with PBS to remove non-adherent platelets, adherent platelets were incubated with 0.1 M citrate buffer containing 5 mM pnitrophenol phosphate and 0.1% Triton X-100 for 1 hour at room temperature. The reaction was stopped by 2 M NaOH and the absorbance was detected at 405 nm.

### 2.11. Platelet aggregation

Platelets were activated with 0.5 U/ml THB or 10 μM ADP at 37°C under stirring condition (1000 rpm). Platelet aggregation was measured using a Lumi Aggregometer (Chrono-Log).

### 2.12. Colt retraction assay

Platelets were added to aggregometer tubes, stimulated with 2 U/mL thrombin, 0.5 mg/mL fibrinogen (341576, Calbiochem, La Jolla, California) and 2 mM CaCl_2_, and incubated at 37°C for 2 hours to observe the contraction every 10 minutes. The clot retraction was captured and quantified using the ratio of clot volume to initial volume.

### 2.13. Hematopoietic stem/progenitor cell differentiation assay

BM was flushed out from femurs by 24 gauge needle into RPMI-1640 medium. The BM cells were washed twice and filtered through 200 mesh nylon net to prepare single cell suspension. Hematopoietic stem/progenitor cells (HSPCs) from BM cells were isolated by a negative selection method with Mouse Hematopoietic Progenitor Cell Isolation Kit (19856, Stem Cell Technologies, Vancouver, Canada). For megakaryocytic differentiation assay, cells were cultured in Stemspan SFEM medium (09600, Stem Cell Technologies) with 10 ng/mL rmIL-3 (213-13, Peprotech, Rocky Hill, New Jersey), 20 ng/mL of rmTPO (78072, Stem Cell Technologies) and rmSCF (78064, Stem Cell Technologies) for 8 days. Megakaryocytic differentiation was assessed by flow cytometry using anti-mouse CD41 antibody. For the myeloid differentiation assay, cells were cultured in Stemspan SFEM medium with 20 ng/mL each of rmIL-3, rmSCF, rmFlt3L (78011, Stem Cell Technologies), and rmG-CSF (78014, Stem Cell Technologies) for 8 days. Myeloid differentiation was assessed by flow cytometry using anti-mouse CD11b and Gr1 antibodies.

### 2.14. Colony forming unit assay

HSPCs were isolated as described above. Colony forming unit (CFU) assays were performed in MethoCult GF M3434 medium (03434, Stem Cell Technologies) according to the manufacture’s protocols. Colony numbers were counted after 10 days of culture under the inverted microscope.

### 2.15. Ploidy analysis

For primary cultured Mks, cells were harvested on day 8 for megakaryocyte ploidy analysis. For cell lines, MEG-01/sh-NC and MEG-01/sh-MXRA7 cells (2 × 10^5^ cells/well) were seeded into 6-well plate and incubated for 72 hours in the presence of 10 ng/mL rhTPO (300-18, Peprotech), then the cells were harvested for analysis. Cells were stained with anti-CD41 antibody 30 minutes at 4°C and washed twice with PBS. Next, the cells were fixed with cold 70% ethanol at −20°C for 4 hours and washed twice with PBS. Finally, the cells were stained with 300 μL PI/RNase Staining Buffer (550825, BD Biosciences, San Diego, California) and incubated for 30 minutes in dark at room temperature before flow cytometry analysis.

### 2.16. Proplatelet formation *assay*

For primary cultured Mks, cells were harvested on day 8 for PPF assay. 24-well plate was coated with 100 μg/mL fibrinogen overnight at 4°C and then blocked with 1% bovine serum albumin for 1 hour at 37°C. Mks were incubated in coated plates to adhere and form proplatelets for 5 hours at 37°C. For cell lines, MEG-01/sh-NC and MEG-01/sh-MXRA7 cells (2 × 10^5^ cells/well) were seeded into 6-well plate and incubated for 72 hours in the presence of 10 ng/mL rhTPO. Proplatelet-forming Mks were captured and counted using light microscopy.

### 2.17. Reverse transcription-quantitative real-time PCR

The total RNA was extracted from BM cells, PBMCs, or MEG-01 cells with RNAiso plus reagent (9109, TaKaRa, Dalian, China), and reverse-transcribed into cDNA with Reverse Transcriptase M-MLV reagent (2641, TaKaRa) according to manufacturer’s instructions. Quantitative real-time PCR (qPCR) was performed using SYBR Premix Ex Taq reagent (rr420, TaKaRa) and carried out on a QuantStudio 3 Real-Time PCR System (Applied Biosystems, Foster, California). The primers used in the study were synthesized by Genewiz Company (Suzhou, China) and listed in Supplemental Table S1, http://links.lww.com/BS/A66. The β-actin gene was used as an internal control and the data were analyzed using 2^−ΔΔCt^ method.

### 2.18. Western blot analysis

Total proteins were extracted from cells with the strong Radio-Immunoprecipitation Assay (RIPA) lysate kit (P0013B, Beyotime), and the concentration of protein samples was determined by BCA method. Proteins were denatured, separated by SDS-PAGE gel and transferred to polyvinylidene difluoride membranes. The membranes were blocked with 5% non-fat powdered milk or 3% BSA in TBST for 2 hours at room temperature and incubated with primary antibodies overnight at 4°C. The primary antibodies used in the study were against MXRA7 (HPA044819, Sigma, St. Louis, MO), AKT (4685), p-AKT (4060), ERK (4695), p-ERK (4370) (all from Cell Signaling Technology, Danvers, Massachusetts), β-Tubulin (HC101, TransGen Biotech, Beijing, China) and GAPDH (KM9002, SUNGENE, Tianjin, China). Following three times washes with TBST, the membranes were incubated with HRP-conjugated goat anti-rabbit IgG (7074) or goat anti-mouse IgG (7076) antibody (Cell Signaling Technology) at room temperature for 1.5 hours. After washing off the unbound antibody with TBST for three times, the protein bands were detected by an enhanced chemiluminescence (ECL) kit (P0018M, Beyotime) using a CLiNX Science Instrument (Shanghai, China). The intensity of the target protein bands was calculated using Image J software.

### 2.19. Cell viability assay

MEG-01/sh-NC and MEG-01/sh-MXRA7 cells (3000 cells/well) were plated into 96-well plate and incubated for 24, 48, and 72 hours to perform Cell Counting Kit-8 (CCK-8) assay. The 10 μL CCK-8 solution (B34302, Biomake, Shanghai, China) was added to each well and incubated for 3 hours. The OD value was measured at 450 nm using a microplate reader (BioTek, Winooski, Vermont).

### 2.20. Cell apoptosis assay

MEG-01/sh-NC and MEG-01/sh-MXRA7 cells (5 × 10^4^ cells/well) were seeded into 24-well plate and incubated for 48 hours. Cell were harvested and stained with Annexin V/PE and 7-AAD (559763, BD Biosciences) according the manufacture’s instruction. The percentage of cell apoptosis was analyzed via flow cytometry.

### 2.21. DMU-212 treatment

In the presence of 10 ng/mL rhTPO, MEG-01/sh-NC and MEG-01/sh-MXRA7 cells (2 × 10^5^ cells/well) were seeded into 6-well plate and treated with or without 50 μM DMU-212 (E2203, Selleck, Shanghai, China). After 72 hours, the cells were harvested for analysis, including the activation of ERK1/2, the expression of CD41 and ploidy analysis.

### 2.22. Immunofluorescence staining

Cells were washed twice and smeared onto 0.1% gelatin coated slides (Thermo Fisher Scientific). After being air-dried at room temperature, the cells were fixed with methanol for 20 minutes and permeabilized with 0.1% Triton X-100 for 25 min. Then, the cells were blocked with 3% BSA in PBS for 20 minutes at room temperature, and incubated with mouse anti-β-Tubulin antibody overnight at 4°C. After washing three times with PBS, the cells were incubated with Alexa Fluor 594 anti-mouse IgG antibody (ab150116, Abcam) for 1 hour at room temperature in dark. Counterstaining with DAPI (C1002, Beyotime) was used to detect nuclei. Microscopy was performed with a Leica TCS SP8 Confocal Microscope (Wetzlar, Germany).

### 2.23. Statistical analysis

Data are presented as mean ± SD. Statistical analyses were performed with Student *t* test (unpaired, two-tailed) and one-way ANOVA with Graphpad prism 9 software (Graphpad, San Diego, California). *P* <.05 was considered as statistically significant (*), <.01 or .001 was shown as ** or ***, respectively.

## 3. RESULTS

### 3.1. Knock-out of MXRA7 decreased the amount of Mks

**Figure 1.**
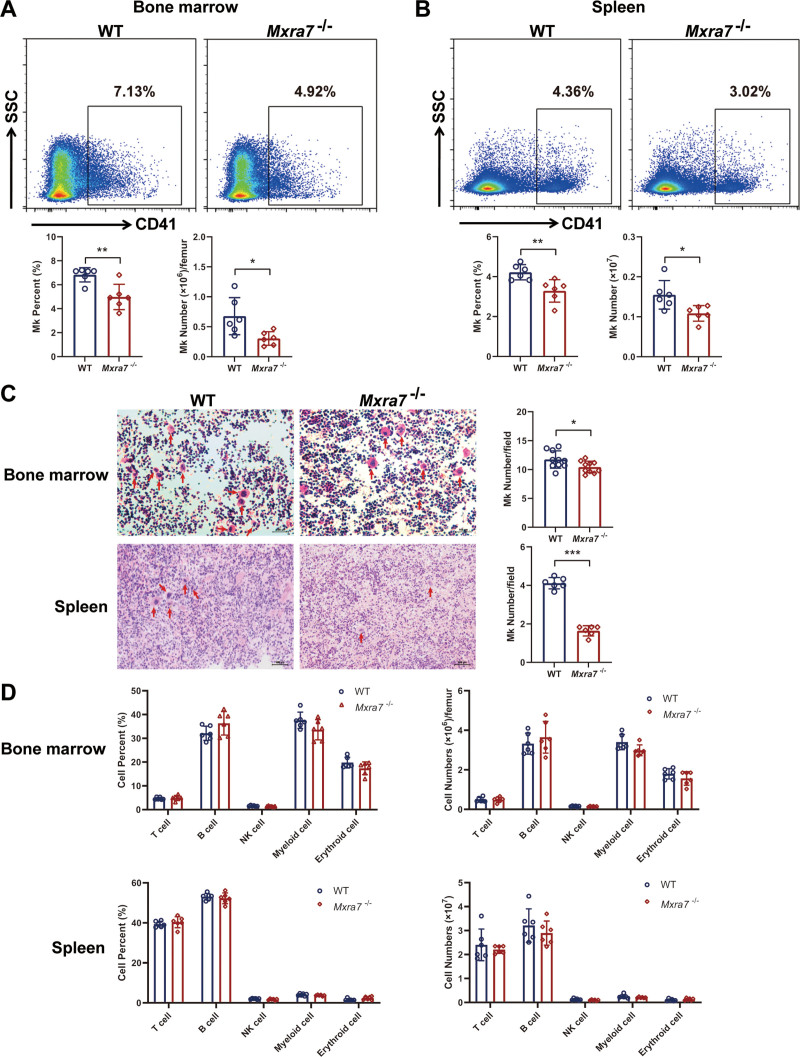
Knock-out of MXRA7 decreased the amount of Mks. (A and B) The percentages of CD41^+^ cells in bone marrow and spleen isolated from WT and *Mxra7*^−/−^ mice (n = 6) were analyzed by flow cytometry. The number of CD41^+^ cells in bone marrow and spleen were also shown. (C) Mks in femur tissues and spleens of WT and *Mxra7*^−/−^ mice were examined by staining the tissue sections with H&E. Red arrows indicate Mk cells. One representative section is shown for each group (original magnification: 100×). The cell number of Mks was determined by six visual fields randomly. (D) The percentages of T, B, NK, myeloid and erythroid cells in bone marrow and spleen of WT and *Mxra7*^−/−^ mice were analyzed by flow cytometry. *P <.05, **P <.01, ***P <.001. Mks = megakaryocytes, WT = wild type.

Mining of public databases showed that MXRA7 was expressed in HSCs as well (Supplemental Fig. S1, http://links.lww.com/BS/A63), suggesting a possibility for MXRA7 to be involved in hematopoiesis. We firstly detected the lineages of hemopoietic cells in the BM, spleen, and peripheral blood from WT and *Mxra7*^−/−^ mice. The result showed that the percentage and number of CD41^+^ cells (Mk) were both decreased in BM cells and splenocytes isolated from *Mxra7*^−/−^ mice (Fig. 1A and B), and the percentage of Mk cells was also decreased in peripheral blood from *Mxra7*^−/−^ mice (Supplemental Fig. S2A, http://links.lww.com/BS/A64). The H&E staining of femurs and spleens from WT and *Mxra7*^−/−^ mice also showed that the number of Mks was reduced in *Mxra7*^−/−^ mice (Fig. 1C). However, knock-out of MXRA7 did not influence other lineages of hemopoietic cells in the BM, spleen, and peripheral blood (Fig. 1D and Supplemental Fig. S2B, http://links.lww.com/BS/A64). These results suggested that knock-out of MXRA7 could decrease the amount of Mks.

### 3.2. MXRA7 affected the amount and function of platelets

**Figure 2.**
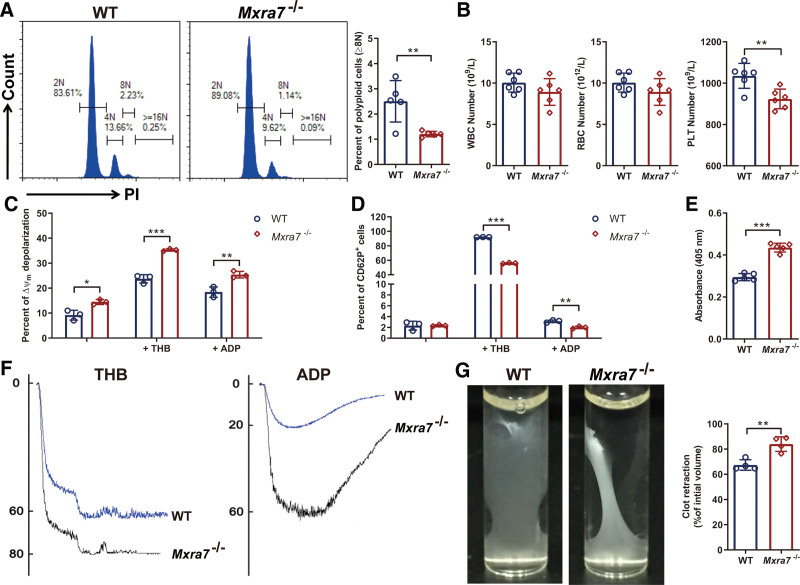
Knock-out of MXRA7 decreased the amount of platelets and affected the function of platelets. (A) The ploidy analysis of Mks in spleen was detected via staining the cells with anti-CD41 antibody and PI/RNase Staining Buffer. The percentage of polyploid cells (≥8N) was analyzed by flow cytometry. (B) WBC, RBC and PLT in peripheral blood of WT and *Mxra7*^−/−^ mice were analyzed. (C and D) Platelets were stimulated with THB or ADP for 30 minutes at room temperature. Mitochondrial membrane potential (ΔΨm) depolarization in platelets was detected by staining with JC-1 and analyzed with flow cytometry. The activation of platelets (CD62P^+^) was analyzed with flow cytometry. (E) The platelets isolated from WT and *Mxra7*^−/−^ mice were incubated on collagen for 1 hour, and the adherent platelets were detected. (F) Platelet aggregation was measured using a Lumi Aggregometer after activated with THB or ADP. (G) Platelets were stimulated with 2 U/mL thrombin, 0.5 mg/mL fibrinogen and 2 mM CaCl_2_. The representative images were captured 1 hour after stimulation. The clot retraction was quantified by the ratio of clot volume to initial volume. *P <.05, **P <.01, ***P <.001. Mks = megakaryocytes, PLT = platelet, RBC = red blood cell, WBC = white blood cell, WT = wild type.

To ensure the role of MXRA7 in the production of platelets from Mks, we analyzed the polyploidy of primary Mks by flow cytometry and compared the peripheral blood of WT and *Mxra7*^−/−^ mice using a hematology analyzer. The results showed that knock-out of MXRA7 reduced the percentage of polyploid cells (≥8N) of Mks in spleen (Fig. 2A), while had no influence on the polyploid cell percentages of Mks in BM and peripheral blood (Supplemental Fig. S3A, http://links.lww.com/BS/A65). Knock-out of MXRA7 also reduced the amount of platelets in peripheral blood (Fig. 2B). There was no significant difference of WBC or RBC between WT and *Mxra7*^−/−^ mice (Fig. 2B). We further made a primary exploration on the function of platelets. The results indicated MXRA7 deficiency up-regulated the apoptosis of platelets (Fig. 2C), and downregulated the activation of platelets simulated with THB or ADP (Fig. 2D). Moreover, MXRA7 deficiency significantly increased the platelet adhesion (Fig. 2E), aggregation (Fig. 2F), and clot retraction (Fig. 2G). However, MXRA7 deficiency did not affect the tail bleeding time and life span of platelets (Supplemental Fig. S3B and C, http://links.lww.com/BS/A65). These results indicated that MXRA7 could not only affect the amount of platelets but also affect the function of platelets.

### 3.3. Knock-out of MXRA7 inhibited Mk differentiation and platelet production

**Figure 3.**
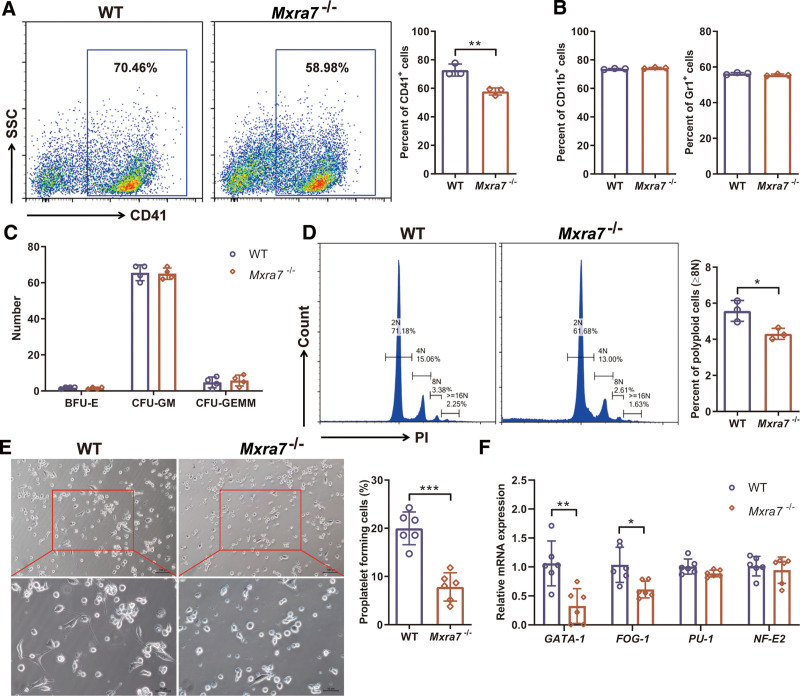
Knock-out of MXRA7 inhibited Mk differentiation and platelet production. (A and B) HSPCs from bone marrow cells of WT and *Mxra7*^−/−^ mice were isolated for megakaryocytic differentiation and myeloid differentiation assays. The percentages of CD41^+^ cells (A), CD11b^+^ and Gr1^+^ cells (B) were analyzed by flow cytometry. (C) HSPCs from bone marrow cells of WT and *Mxra7*^−/−^ mice were isolated for colony forming unit assays. Colony numbers were counted after 10 days of culture. (D) The ploidy analysis of the cultured Mks was detected via staining the cells with anti-CD41 antibody and PI/RNase Staining Buffer. The percentage of polyploid cells (≥8N) was analyzed by flow cytometry. (E) The PPF of the culture Mks was captured and the percentage of cells of proplatelet forming cells was calculated. (F) Expression of *GATA-1*, *FOG-1*, *PU-1*, and *NF-E2* mRNAs in bone marrow cells from WT and *Mxra7*^−/−^ mice was detected by RT-qPCR. *P <.05, **P <.01, ***P <.001. HSPC = hematopoietic stem/progenitor cell, Mks = megakaryocytes, PPF = proplatelet formation, WT = wild type.

To study the effect of MXRA7 on Mk differentiation, we isolated the HSPCs from the BM cells of WT and *Mxra7*^−/−^ mice. Knock-out of MXRA7 reduced the percentage of Mks (CD41^+^) differentiated from HSPCs (Fig. 3A). While knock-out of MXRA7 had no effect on the differentiation of myeloid cells (CD11b^+^, Gr1^+^) from HSPCs (Fig. 3B), and did not change the morphology and number of BFU-E, CFU-GM, and CFU-GEMM (Fig. 3C). Then, we found that knock-out of MXRA7 could also decrease the percentage of polyploid cells (≥8N) of the cultured Mks (Fig. 3D). In addition, the percentage of proplatelet forming cells was reduced by knock-out of MXRA7 (Fig. 3E). To further investigate the cause of decreasing Mks and platelets, we evaluated the expression of transcription factors related to megakaryocyte maturation and differentiation in BM cells via RT-qPCR. The expression of *GATA-1* and *FOG-1* was lower in *Mxra7*^−/−^ mice than that in WT mice (Fig. 3F). Therefore, the results demonstrated that MXRA7 deficiency inhibited Mk differentiation and platelet production.

### 3.4. MXRA7 influenced the characteristics of MEG-01 cells

**Figure 4.**
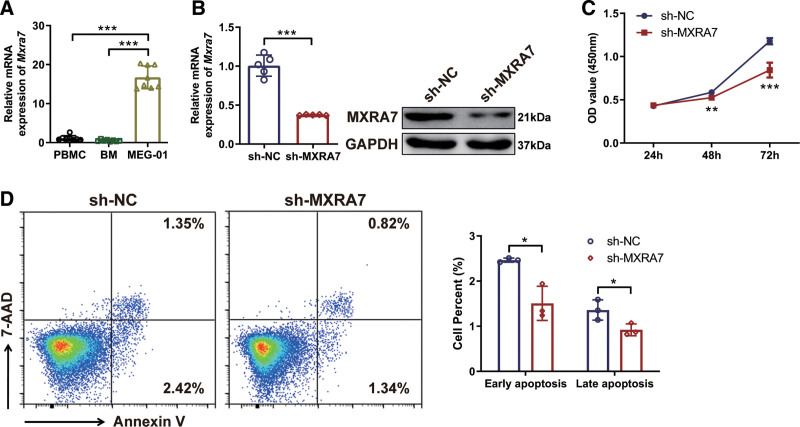
Knock-down of MXRA7 influenced the characteristics of MEG-01 cells in vitro. (A) The mRNA expression of MXRA7 in PBMCs, BM cells, and MEG-01cells was detected by RT-qPCR. (B) The mRNA and protein expression of MXRA7 in MEG-01/sh-NC and MEG-01/sh-MXRA7 cells were detected by RT-qPCR and western blot, respectively. (C) The cell viability of in MEG-01/sh-NC and MEG-01/sh-MXRA7 cells was compared with colorigenic method. (D) The cell apoptosis of MEG-01/sh-NC and MEG-01/sh-MXRA7 cells were detected with flow cytometry analysis. Early apoptosis referred to Annexin-V^+^ 7-AAD^−^ cells, while late apoptosis referred to Annexin-V^+^ 7-AAD^+^ cells. *P <.05, **P <.01, ***P <.001. BM = bone marrow, PBMC = peripheral blood mononuclear cell, RT-qPCR = reverse transcription-quantitative real-time PCR.

To investigate the mechanism of MXRA7 in the differentiation of Mks, we performed in vitro assays using a megakaryoblastic cell line. By measuring MXRA7 expression in PBMCs, BM cells and MEG-01 cells, we found that MXRA7 mRNA expression was higher in MEG-01 cells than in PBMCs and BM cells (Fig. 4A). Therefore, we constructed MXRA7 knock-down MEG-01 cells to study the function of MXRA7 in megakaryocyte proliferation and differentiation, and the expression of MXRA7 was down-regulated in sh-MXRA7 cells detected by RT-qPCR and western blot analysis (Fig. 4B). We found that knock-down of MXRA7 inhibited cell viability of MEG-01cells via the CCK-8 assay (Fig. 4C). Moreover, knock-down of MXRA7 reduced the apoptosis of MEG-01 cells (Fig. 4D). These results indicated that MXRA7 affected the characteristics of megakaryoblastic cells.

### 3.5. Knock-down of MXRA7 inhibited the differentiation of MEG-01 cells

**Figure 5.**
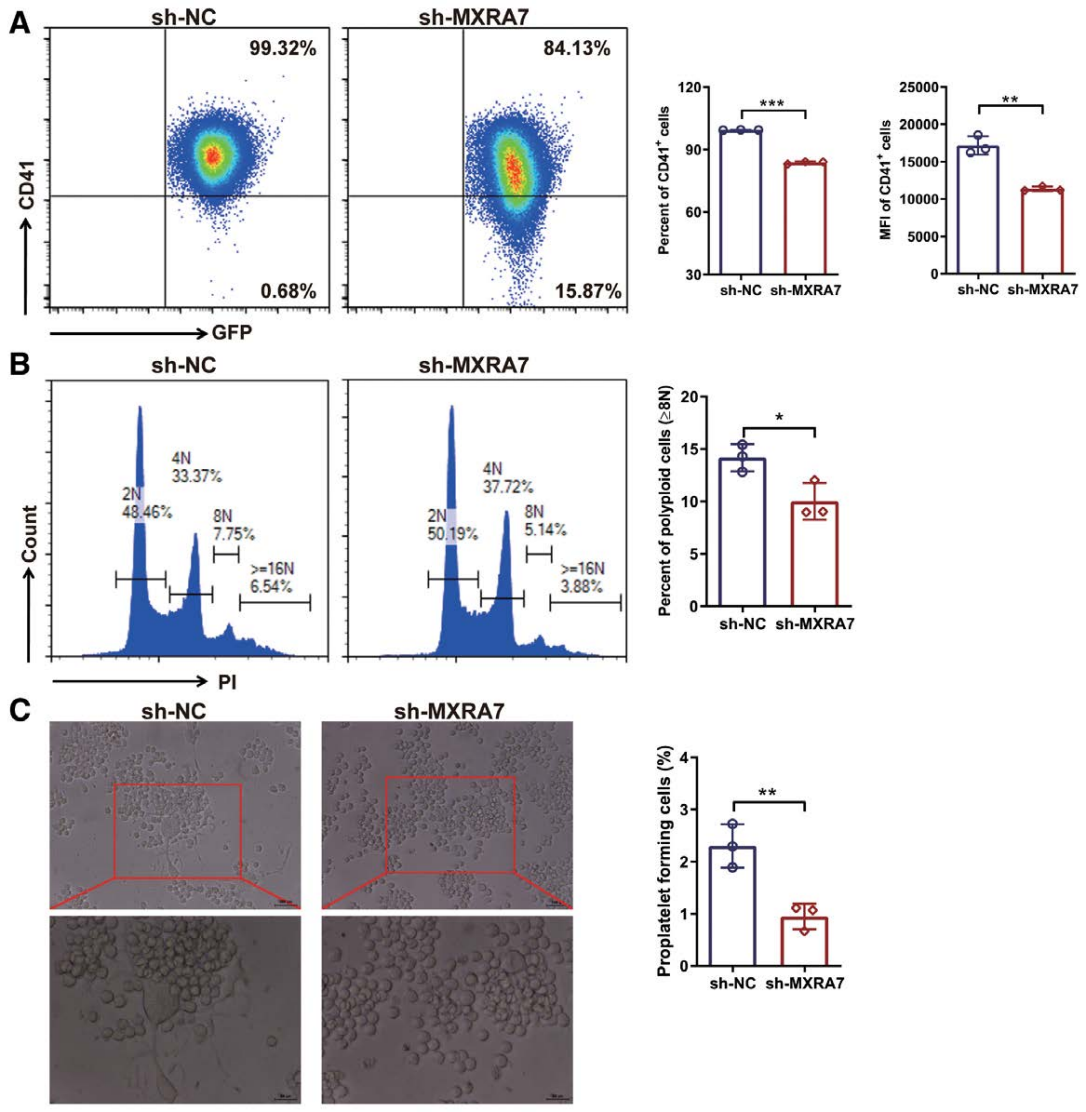
Knock-down of MXRA7 inhibited Mk differentiation in vitro. MEG-01/sh-NC and MEG-01/sh-MXRA7 cells were incubated for 72 hours in the presence of 10 ng/mL rhTPO. (A) The percentage and mean fluorescence (MFI) of CD41^+^ cells were analyzed by flow cytometry. (B) The percentage of polyploid cells (≥8N) was analyzed by flow cytometry via staining the cells PI/RNase Staining Buffer. (C) Typical PPF was captured and the percentage of cells with PPF was calculated. *P <.05, **P <.01, ***P <.001. Mk = megakaryocyte, PPF = proplatelet formation.

To further investigate the effect of MXRA7 on the differentiation of Mks, we induced the differentiation of MEG-01 cells and proplatelet formation (PPF) in the presence of TPO in vitro. Knock-down of MXRA7 decreased the percentage and mean fluorescence intensity of CD41^+^ cells (Fig. 5A). Moreover, knock-down of MXRA7 also reduced the percentage of polyploid cells (≥8N) (Fig. 5B) and inhibited PPF (Fig. 5C). These results demonstrated that knock-down of MXRA7 could inhibit the differentiation of MEG-01 cells and reduce PPF.

### 3.6. Knock-down of MXRA7 inhibited the ERK/MAPK signaling pathway and decreased the expression of β-tubulin

**Figure 6.**
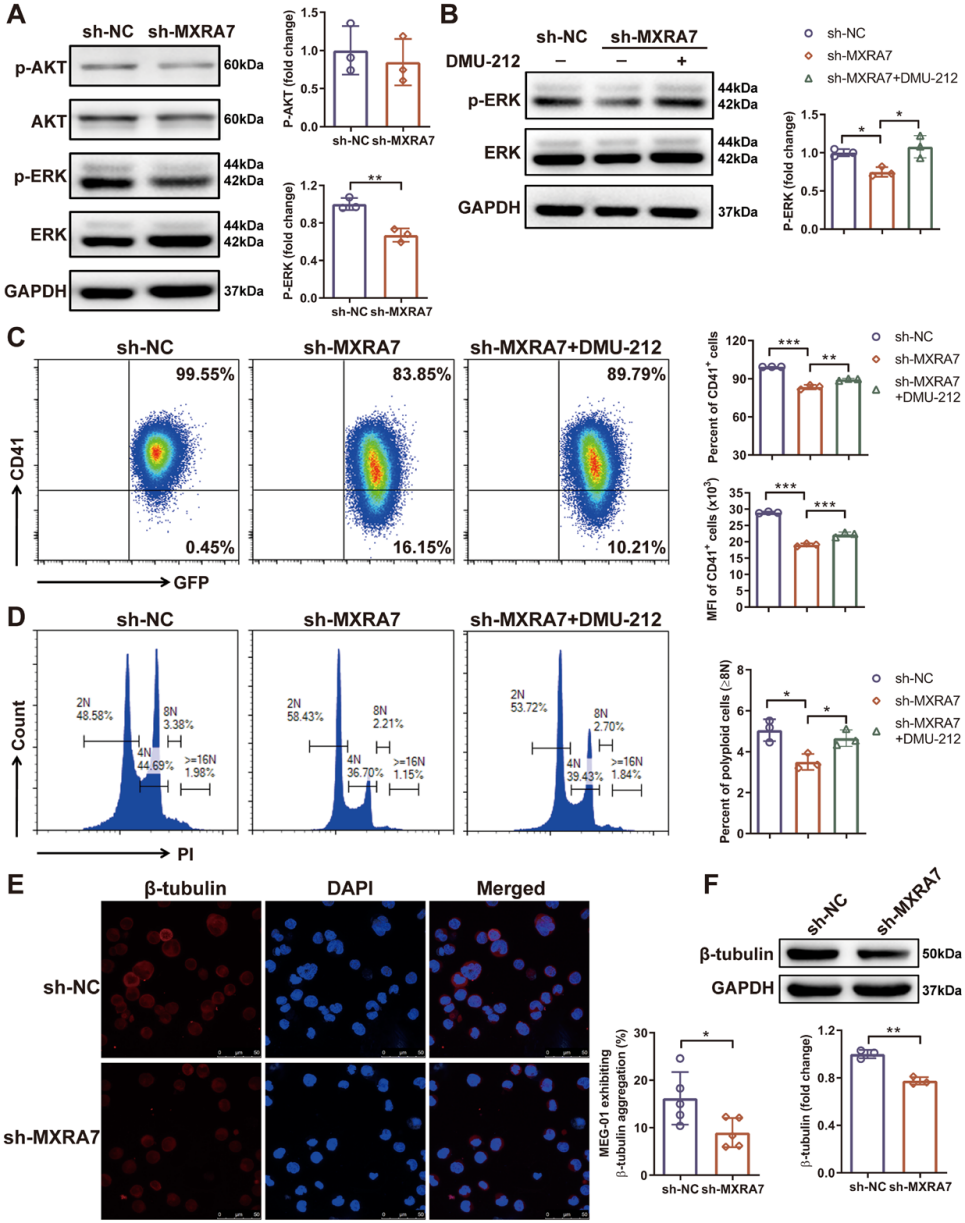
Knock-down of MXRA7 inhibited the ERK/MAPK signaling pathway and decreased the expression of β-tubulin. MEG-01/sh-NC and MEG-01/sh-MXRA7 cells were incubated for 72 hours in the presence of 10 ng/mL rhTPO. (A) Proteins were extracted from cell lines for western blot analysis of p-AKT, AKT, p-ERK and ERK. (B) Proteins were extracted from cell lines treated with or without DMU-212 for western blot analysis of p-ERK and ERK. (C and D) Treated with or without DMU-212, the percentage and mean fluorescence (MFI) of CD41^+^ cells, and the percentage of polyploid cells (≥8N) were analyzed by flow cytometry. The expression of β-Tubulin was detected by immunofluorescence staining (E) and western blot (F). *P <.05, **P <.01, ***P <.001.

To elucidate the mechanism of MXRA7 in Mk differentiation, we detected the signaling pathways involved in this process. TPO and its MK-specific receptor c-Mpl activate multiple signaling pathways which play critical roles in the process of megakaryopoiesis, including ERK/MAPK, PI3K/Akt, and JAK/STAT pathways.^14–16^ We found that knock-down of MXRA7 inhibited the expression of phosphorylated ERK1/2, but did not affect the phosphorylation of Akt (Fig. 6A). To confirm whether MXRA7 affected Mk differentiation through ERK/MAPK signaling pathway, we performed the rescue assay using DMU-212, an activator of ERK1/2 protein.^17^ We found that DMU-212 could upregulate the phosphorylation of ERK1/2. Moreover, DMU-212 treatment could partially rescue the expression of CD41 (Fig. 6C), and increase the percentage of polyploid cells (≥8N) (Fig. 6D), which were downregulated by MXRA7 silencing.

At the end of the maturation of Mks, the PPF requires cytoskeletal changes, including changes in microtubule and actin organization.^18^ Microtubules can facilitate elongating the shaft and tips of proplatelets.^19–21^ We demonstrated that knock-down of MXRA7 could decrease β-tubulin expression (Fig. 6 E and F). Therefore, these results suggested that MXRA7 affected megakaryocyte differentiation and platelet production mainly through mediating the ERK/MAPK signaling pathway and the expression of β-tubulin.

## 4. DISCUSSION

This study enriched our knowledge about MXRA7, which had been neglected since its nomenclature in 2002 and gained attention only recently.^22^ In brief, MXRA family consists of eight numbers, MXRA1-MXRA8, which was co-expressed with genes involved in cell adhesion or matrix remodeling (matrix metalloproteinases, collagens, bone morphogenic proteins, etc).^6^ MXRA7 had been mentioned in running in some studies, which suggested increased expression of MXRA7 in several marrow malignancies as summarized.^22^

In this study, we firstly used *Mxra7*^−/−^ mice and WT mice to investigate the role of MXRA7, and found that knock-out of MXRA7 decreased the amount of Mks, but did not affect other lineages of hemopoietic cells In vivo (Fig. 1). Knock-out of MXRA7 also reduced the amount of platelets. Moreover, MXRA7 deficiency increased the apoptosis of platelets and inhibited the activation of platelets. MXRA7 deficiency also increased platelet adhesion, aggregation and clot retraction (Fig. 2). In in vitro experiments, knock-out of MXRA7 could inhibit HSPCs differentiate to Mks, decrease the percentage of polyploid cells (≥8N) of the cultured Mks, and reduce the PPF (Fig. 3). These results indicated MXRA7 not only affected Mk differentiation and platelet production, but also affected the function of platelets. The transcription factors play important roles in megakaryopoiesis and platelet biogenesis, such as GATA-1, NF-E2, and so on.^23^ We found that knock-out of MXRA7 down-regulated the mRNA expression of GATA-1 and FOG-1. GATA-1 which interacts with its cofactor FOG-1, is crucial for Mk differentiation.^24^ Therefore, MXRA7 may regulate differentiation of Mks through mediating the expression of GATA-1 and FOG-1.

To further confirm the role of MXRA7 in human Mks and investigate the mechanism of MXRA7 in the Mk differentiation, we performed the assays using a human megakaryoblastic cell line, MEG-01. The results showed that MXRA7 affected the characteristics of megakaryoblastic cells. Although knockdown of MXRA7 decreased cell apoptosis, but still inhibited cell viability, possibly caused by inhibition of cell proliferation (Fig. 4). The role of megakaryocyte apoptosis in platelet production remains inconclusive,^25,26^ there are still further investigations to be conducted regarding the role of intrinsic and extrinsic apoptosis pathways in megakaryocyte and platelet production. Importantly, knock-down of MXRA7 also inhibited the differentiation of MEG-01 cells, and reduced the PPF (Fig. 5). There are multiple secreted factors regulate megakaryocyte differentiation and platelet production. TPO functions as the major regulator that acts at early and late stages of megakaryopoiesis, and activates multiple signaling pathways, including ERK/MAPK and PI3K/Akt pathways.^14–16^ And we demonstrated that knock-down of MXRA7 inhibited the expression of phosphorylated ERK1/2. The rescue assay of ERK1/2 activation was further indicated the effect of MXRA7 on Mk function mostly through the ERK/MAPK signaling pathway. Moreover, knock-down of MXRA7 could decrease the expression of β-tubulin, which facilitates platelet production (Fig. 6).

In summary, our data described above demonstrated that MXRA7 was involved in Mk differentiation, platelet production, and function. This added MXRA7 to the list of genes involved in hemopoiesis. To be more specific, MXRA7 might incur its functions in hematopoiesis or megakaryopoiesis via the process mediated by ECM, which might be through mediating the ERK/MAPK pathway and β-tubulin expression. Our data indicated the potential significance of MXRA7 in Mk differentiation in mice and human megakaryoblastic cell line. Public database demonstrated that MXRA7 gene was conservative among vertebrate species, including mice and human.^22^ In general, these data proposed that MXRA7 may be a new target for the treatment of platelet-related diseases, and much more investigations are guaranteed to dissect the mechanisms of MXRA7 modulation in Mk differentiation and platelet production or in overall pathology of the platelet-related diseases. Public data in platforms like GEO and Bloodspot showed that MXRA7 was expressed highly in ALL and acute myeloid leukemia (AML). MXRA7 was among an 86-probe-set with prognostic significance for cytogenetically normal AML.^27^ Therefore, more extensive investigations are deserved to fully understand the role of MXRA7 in physiological or pathological hematopoiesis.

## ACKNOWLEDGMENTS

This work was supported by grants from the National Natural Science Foundation of China (81600076, 81271050, 82070186), Suzhou Science and Technology Program Project (SKY2022043), and the Innovation special project of science and technology of Jiangyin (JY0603A021014210012).

## AUTHOR CONTRIBUTIONS

D.L. and Y.W. designed the research and wrote the manuscript; Z.S., B.W., Y.S., K.M. and T.W. performed the experiments and collected the data; Z.S. and B.W. analyzed and interpreted data. All authors approved the final version of the manuscript.

## Supplementary Material








