# Supplementary material for: MXRA7 is involved in megakaryocyte differentiation and platelet production
Source: Blood Sci. 2023 Jul 5;5(3):160–9. doi: 10.1097/BS9.0000000000000167 (PMC10400050; doi:10.1097/BS9.0000000000000167)
Supplement: Supplementary file 1 [file bs9-5-160-s001.pdf]

Table S1. Primer sequences used for RT-qPCR.

Gene	Primer sequence
GATA-1 (mouse)	Forward: 5′-ACTCCTCCTGGTCCGAATGC-3′
	Reverse: 5′-GCCCCTAGACCAGGAAAATCC-3′
FOG-1 (mouse)	Forward: 5′-GGACTGCTATATGTGCGCCT-3′
	Reverse: 5′-ACCAGATCCCGCAGTCTTTG-3′
PU-1 (mouse)	Forward: 5′-ACCTTCCAGTTCTCGTCC-3′
	Reverse: 5′-CTGTCTTGCCGTAGTTGC-3′
NF-E2 (mouse)	Forward: 5′-TTGGAATCCGCCACAGGTTG-3′
	Reverse: 5′-ACCCTGCAGCTCAGTAATGG-3′
β-actin (mouse)	Forward: 5′-GCTCCTAGCACCATGAAGAT-3′
	Reverse: 5′-GTGTAAAACGCAGCTCAGTA-3′
MXRA7 (human)	Forward: 5′-GCTGAGGGGAAACCAGTACA -3′
	Reverse: 5′-CGGACATCTCGCCAAACG -3′
β-actin (human)	Forward: 5′-CTCACCATGGATGATGATATCGC-3′
	Reverse: 5′-AGGAATCCTTCTGACCCATGC-3′
