# Supplementary material for: MXRA7 is involved in megakaryocyte differentiation and platelet production
Source: Blood Sci. 2023 Jul 5;5(3):160–9. doi: 10.1097/BS9.0000000000000167 (PMC10400050; doi:10.1097/BS9.0000000000000167)
Supplement: Supplementary file 2 [file bs9-5-160-s002.pdf]

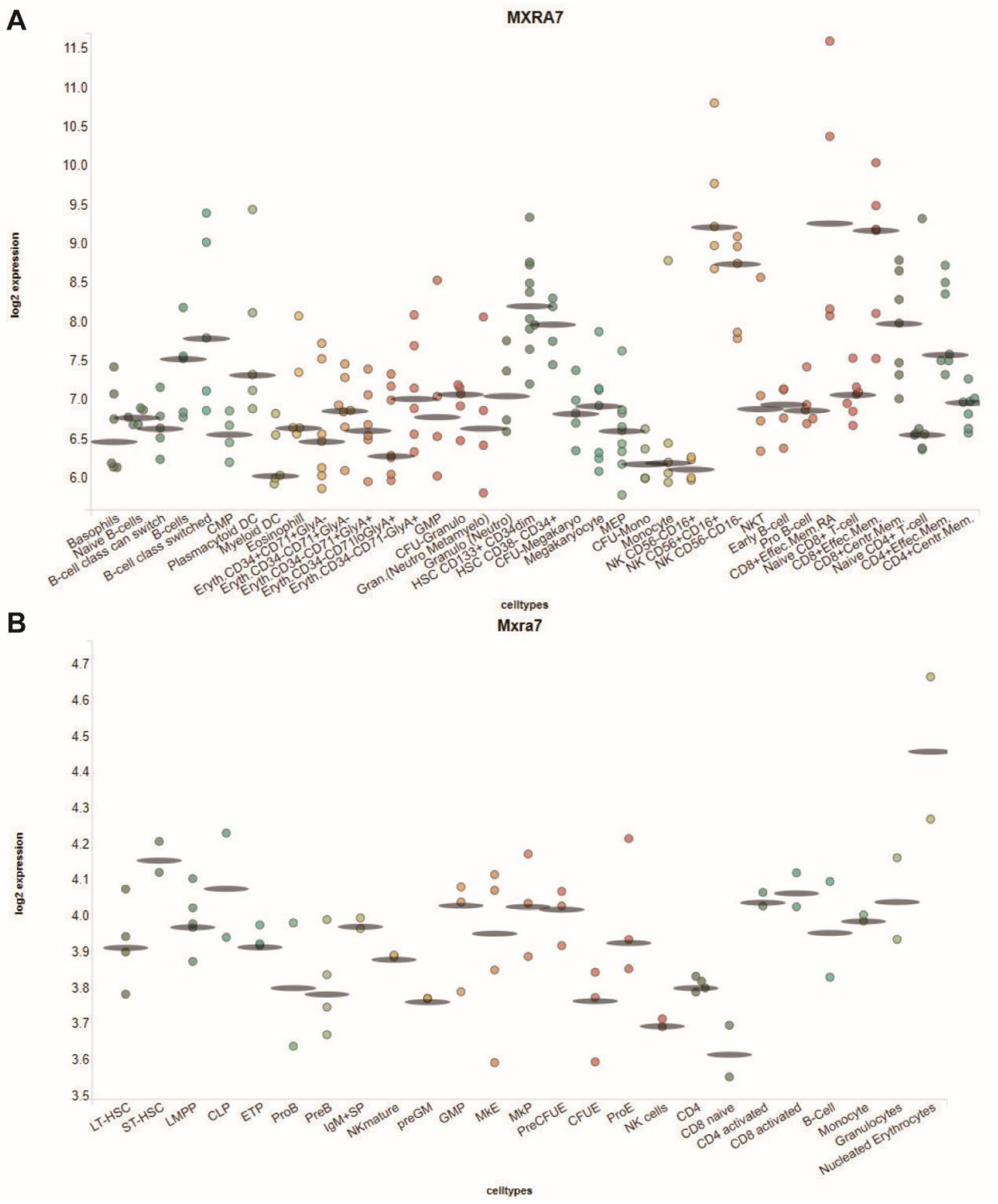

Figure S1. The expression of MXRA7 mRNA in databank of Bloodspot program. The atlases were the direct outputs of Bloodspot program for "Normal human hematopoiesis (DMAP)" (A) and "Mouse normal hematopoietic system" (B). Y axis values were the absolute expression levels in log2 formats for the "Max probe", herein 212509_s_at (A) and 1453855_at (B). The grey spindles indicated the median value of each group. Details of data and rationale for producing this plot were available from the Bloodspot website.
