# Supplementary material for: MXRA7 is involved in megakaryocyte differentiation and platelet production
Source: Blood Sci. 2023 Jul 5;5(3):160–9. doi: 10.1097/BS9.0000000000000167 (PMC10400050; doi:10.1097/BS9.0000000000000167)
Supplement: Supplementary file 3 [file bs9-5-160-s003.pdf]

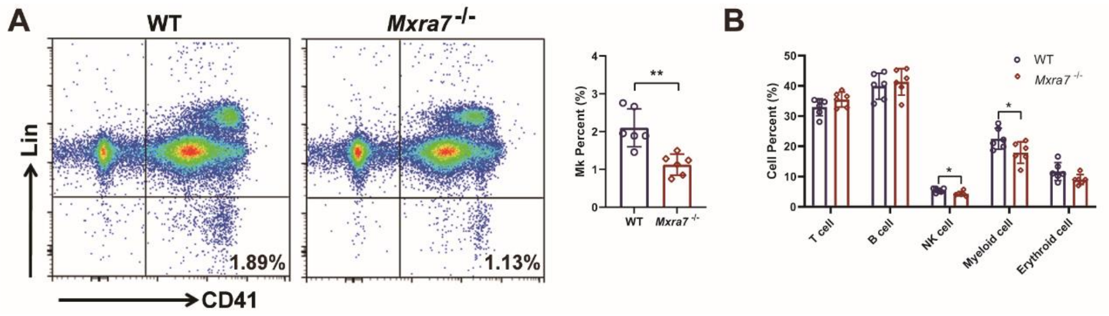

Figure S2. The lineages of hemopoietic cells in peripheral blood.

(A) The percentage of Mk cells in peripheral blood of WT and *Mxra7*$^{-/-}$ mice were analyzed by flow cytometry. (B) The percentages of T, B, NK, myeloid and erythroid cells in peripheral blood of WT and *Mxra7*$^{-/-}$ mice were analyzed by flow cytometry.
