# Supplementary material for: MXRA7 is involved in megakaryocyte differentiation and platelet production
Source: Blood Sci. 2023 Jul 5;5(3):160–9. doi: 10.1097/BS9.0000000000000167 (PMC10400050; doi:10.1097/BS9.0000000000000167)
Supplement: Supplementary file 4 [file bs9-5-160-s004.pdf]

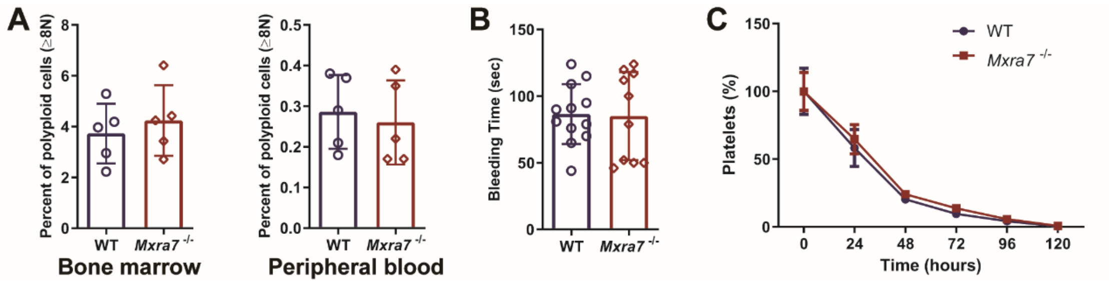

Figure S3. The role of MXRA7 in platelet production and function.

(A) The ploidy analysis of Mks in bone marrow and peripheral blood were detected by flow cytometry. The percentage of polyploid cells (≥8N) was analyzed. (B) 5 mm of the tail tip was cut from WT and *Mxra7*$^{-/-}$ mice, and the tail was immediately immersed in NaCl solution. The bleeding time was recorded. (C) The platelets were isolated from WT and *Mxra7*$^{-/-}$ mice and labeled with calcein-AM, then the platelets were injected into WT mice by tail vein. The anticoagulant whole blood was collected and stained with anti-CD41 antibody. The percentage of platelets labeled with calcein-AM in the total platelets was analyzed with flow cytometry.
